# Recovery of Two Replication-Competent Canine Distemper Viruses That Separately Express Dabie Bandavirus Gn and Gc

**DOI:** 10.3389/fvets.2022.845845

**Published:** 2022-03-31

**Authors:** Jiahui Lin, Yuehua Li, Liangpeng Lyu, Qianqian Wang, Hui Zhang, Bo Ni, Fuxiao Liu

**Affiliations:** ^1^College of Veterinary Medicine, Qingdao Agricultural University, Qingdao, China; ^2^China Animal Health and Epidemiology Center, Qingdao, China; ^3^Qingdao Workstation of Animal Husbandry, Qingdao, China

**Keywords:** severe fever with thrombocytopenia syndrome, Dabie bandavirus, canine distemper virus, Gn and Gc, recombinant virus, reverse genetics

## Abstract

Severe fever with thrombocytopenia syndrome (SFTS) is an emerging tick-borne zoonosis with a high mortality rate in humans. Additionally, dogs are frequently reported to be infected with this disease. There has been no commercially available vaccine for humans and animals as yet. The SFTS is caused by Dabie bandavirus (DBV), formerly known as SFTS virus. The DBV is now classified into the genus *Bandavirus* in the family *Phenuiviridae*. DBV Gn and Gc can induce specific immune responses *in vivo*. In this study, we used reverse genetics technique to construct two recombinant canine distemper viruses (rCDVs), rCDV-Gn and -Gc, which could express Gn and Gc *in vitro*, respectively. Both of the recombinants, derived from a common parental CDV, were independently subjected to twenty serial passages in cells for Sanger sequencing. Neither point mutation nor fragment deletion was found in the Gn open reading frame (ORF), whereas the rCDV-Gc showed a nonsynonymous mutation (A157C) in the Gc ORF, correspondingly resulting in a mutation of amino acid (T53P) in the Gc. Growth curve of the rCDV-Gc almost coincided with that of a wild-type CDV, but exhibited a significant difference from that of the rCDV-Gn. Much research remains to be performed to demonstrate whether both recombinants are able of inducing specific immune responses *in vivo*.

## Introduction

Severe fever with thrombocytopenia syndrome (SFTS) is an emerging infectious disease, caused by Dabie bandavirus (DBV), formerly known as SFTS virus. This disease was initially reported in Central China in 2009. Its clinical signs include fever, thrombocytopenia, gastrointestinal symptoms and leukocytopenia in patients. There is an unusually high initial case fatality rate of 30% (1). In recent years, this disease has raised serious public health concerns, especially in China (2). The SFTS is a tick-borne zoonosis. The DBV can rapidly evolve by gene mutation, reassortment and homologous recombination in ticks and reservoir hosts (3). More recently, it was frequently reported that non-human animals, especially dogs, were infected by DBV, or were diagnosed with DBV antibody-positive (4–9). Dog-to-human transmission of DBV can even occur through manual de-ticking of domestic dogs (10). Specific treatment of SFTS is unavailable now. Development of veterinary vaccines would be one of the most effective ways to protect companion dogs from SFTS, thereby interrupting a potential route of dog-to-human transmission.

The DBV belongs to the genus *Bandavirus* in the family *Phenuiviridae* of the order *Bunyavirales*. Its genome is segmented into three pieces: L, M and S segments. The M segment encodes a membrane protein precursor that matures into two glycoproteins, Gn and Gc, embedded within the viral envelope. Bunyaviral Gn and Gc can induce specific immune responses *in vivo* (11–14). Different virus-vectored vaccines have been reported to be capable of inducing DBV-specific immune responses (15–18). For example, Dong et al. (19) constructed a live-attenuated recombinant vesicular stomatitis virus that could express the DBV Gn/Gc glycoproteins. Single-dose vaccination with it was demonstrated to elicit complete protection in mice from DBV infection (18). More recently, Tian et al. (15) reported that Gn-expressing recombinant rabies virus conferred protective immune responses in mice.

Canine distemper virus (CDV), also known as canine morbillivirus, causes a highly contagious disease, canine distemper, which affects a wide variety of domestic and wild carnivores (19). This virus is classified into the genus *Morbillivirus* in the family *Paramyxoviridae*. Typical CDV virions are enveloped and pleomorphic particles. The viral genome is a single-stranded, linear RNA with negative polarity. Wild-type CDV possesses a 15,690-nt-long genome, following the “rule of six”, necessary for efficient replication between genome and antigenome (20). The CDV genome contains six transcriptional units, separately coding for six structural proteins, namely N, P, M, F, H and L proteins. Six open reading frames (ORFs) are separated by untranslated regions with variable lengths.

Virulence-attenuating CDV strains have been broadly used to produce commercially available vaccines against canine distemper. Moreover, these strains are potential vectors for delivering foreign antigens to induce protective immunities against canine distemper and other diseases (21), such as leishmaniasis (22) and rabies (23). Unfortunately, there has been no report concerning CDV-vectored vaccines against DBV as yet. We had developed one virulence-attenuating strain (CDV QN strain) previously, and more recently, we constructed its reverse genetics platform for expressing foreign antigens (24, 25). Considering anti-DBV vaccines unavailable for dogs, we rescued two recombinant CDVs in the present study. These two recombinants were demonstrated to be able to express separately DBV Gn and Gc *in vitro*.

## Materials and Methods

### Cells, Virus and Plasmids

Two cell lines, BSR-T7/5 and Vero-Dog-SLAM (VDS), were kindly provided by the China Animal Health and Epidemiology Center, and cultured at 37°C with 5% CO_2_ in Dulbecco's modified Eagle's medium (DMEM), supplemented with fetal bovine serum (VivaCell, Shanghai, China), penicillin (100 U/mL), streptomycin (100 μg/mL), amphotericin B (0.25 μg/mL) and G418 (500 μg/mL). The wild-type CDV (wt-CDV), QN strain, was cultured in VDS cells. The QN strain-based reverse genetics system had been established previously, mainly containing four plasmids, namely one full-length cDNA clone of recombinant CDV that expressed a foreign protein, and three helper plasmids (pCAGGS-N, pCAGGS-P and pCAGGS-L).

### Construction of Two Recombinant CDV cDNA Clones

**Figure 1:**
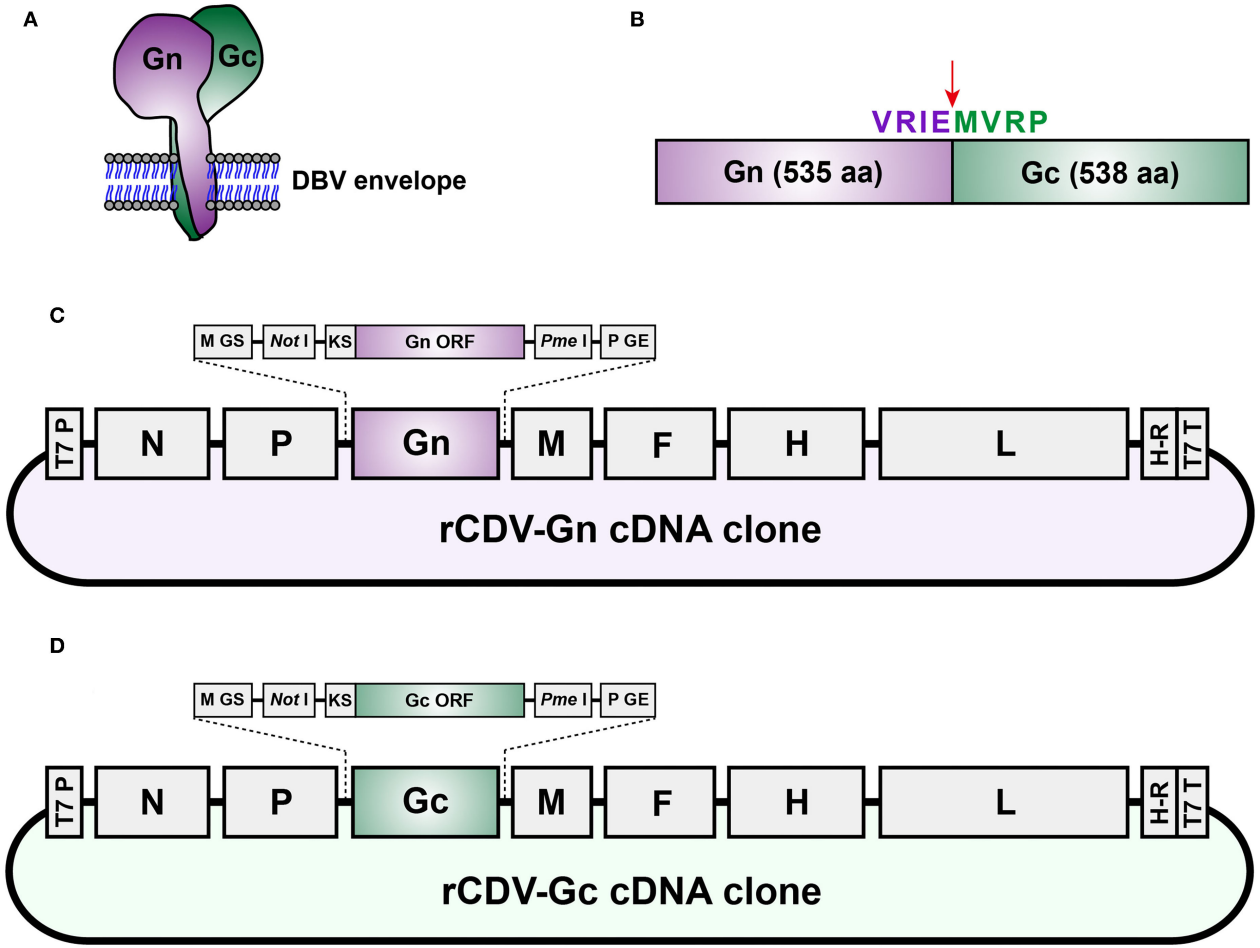
Schematic representations of DBV Gn-Gc heterodimer, glycoprotein precursor, rCDV-Gn cDNA clone and rCDV-Gc cDNA clone. DBV spike is a Gn-Gc heterodimer, embedded within viral envelope **(A)**. DBV glycoprotein precursor composed of Gn and Gc **(B)**. The cleavage site is marked with a red arrow. rCDV-Gn cDNA clone **(C)** and rCDV-Gc cDNA clone **(D)**. T7 P, T7 promoter; GS, gene start; GE, gene end; KS, Kozak sequence; H-R, hepatitis delta virus ribozyme; T7 T, T7 terminator.

Mature Gn and Gc, embedded within the DBV envelope (Figure 1A), are derived from the same precursor (Figure 1B). Two recombinant CDV cDNA clones (rCDV-Gn and -Gc cDNA clones) were schematically shown in Figures 1C,D. They were separately flanked by the T7 promoter and a fusion sequence of hepatitis delta virus ribozyme-T7 terminator at their 5′ and 3′ ends, respectively. In order to improve protein expression, Gn and Gc ORFs (Genbank access No.: MT236316) were optimized for codon usage bias in dogs using an online codon-optimizing tool (https://www.vectorbuilder.cn/tool/codon-optimization.html), followed by chemical synthesis. These two codon-optimizing sequences were independently subcloned into the *Not* I/*Pme* I sites of another CDV cDNA clone (25) *via* homologous recombination using the In-Fusion® Kit (Takara, Dalian, China) according to the manufacturer's instruction. Two recombinant cDNA clones (Figures 1C,D) were subjected to Sanger sequencing for confirming their identities, followed by plasmid extraction using the HighPure Maxi Plasmid Kit (TIANGEN, Beijing, China) according to the manufacturer's instruction.

### Recovery of Two Recombinant CDVs

Two recombinant CDVs, rCDV-Gn and -Gc, were rescued from their individual cDNA clones. Briefly, BSR-T7/5 cells were seeded into a 12-well plate for culturing at 37°C in an incubator. To rescue recombinant CDV, a cell monolayer at 70% confluency was co-transfected with either of the cDNA clones (2.0 μg/well), pCAGGS-N (1.0 μg/well), pCAGGS-P (0.5 μg/well) and pCAGGS-L (0.5 μg/well) using Lipofectamine 2000 (Thermo Fisher, Carlsbad, the USA) according to the manufacturer's instruction. Two plasmid-co-transfected cell monolayers were digested with trypsin at 72 h post transfection (hpt), and then separately co-cultivated with VDS cells in two T25 flasks. Recombinant viruses would be rescued from their individual cDNA clones, and then undergo budding from membranes of BSR-T7/5 cells for further infecting VDS cells. The rescued viruses were subjected to serial blind passages in VDS cells.

### RT-PCR Detection

The rCDV-Gn- and-Gc-infected cell cultures were collected at passage-10 (P10). Total RNAs were extracted from the cell cultures, and then served as templates for RT-PCR detection using the PrimeScript™ High Fidelity One Step RT-PCR Kit (Takara, Dalian, China). The forward (5′-TCAAGAGTATTACTCATGCTTAA-3′) and reverse (5′-TCGAAGTCGTACACCTCAGTCAT-3′) primers targeted sites at the P and M ORFs, respectively. The RT-PCR underwent 45°C for 10 min, 94°C for 2 min and then 30 cycles at 98°C (10 s), 55°C (15 s) and 68°C (20 s). The extracted total RNAs were also subjected to PCR for detecting cDNA residues using the same primer pair. The PCR reaction contained 2 × PrimeSTAR Max Premix (Takara, Dalian, China) and underwent 30 cycles at 98°C (10 s), 55°C (10 s) and 72°C (10 s). Both RT-PCR and PCR products were analyzed through agarose gel electrophoresis. Two RT-PCR products were extracted from gels for Sanger sequencing.

### Indirect Immunofluorescence Assay

IFA was carried out to confirm successful rescue of recombinant CDVs, as described previously (25). In brief, two VDS cell monolayers at 90% confluency were independently inoculated with the P15 rCDV-Gn and -Gc for incubation at 37°C. At 24 h post inoculation (hpi), cell monolayers were fixed with 4% paraformaldehyde for at least 30 min, and then washed four times with PBS for further cellular permeation with 0.4% Triton X-100 for 30 min, followed by washing with PBS thrice. Cell monolayers were blocked in blocking solution at 37°C for 1 h, and then incubated with the anti-CDV monoclonal antibody (MAb) (Lvdu, Binzhou, China) at 37°C for 2 h, followed by washing with PBS thrice. Subsequently, cell monolayers were incubated with the Alexa Fluor® 555 conjugate (Thermo Fisher, Waltham, MA, the USA) at 37°C for 1 h, followed by washing with PBS thrice. After coating with 90% glycerin, cell monolayers were observed under a fluorescence microscope.

### Mass Spectrometry

Gn and Gc expressions were analyzed by mass spectrometry (MS) at the Shanghai Bioprofile Biotechnology Co., Ltd (Shanghai, China), as described previously (26). In brief, rCDV-Gn- and -Gc-infected cell cultures were harvested at P10 for inactivation by 0.1% formalin at 4°C for 48 h. Proteins of inactivated samples were digested by a method of filter-aided sample preparation (27). Liquid chromatography linked to tandem mass spectrometry was performed on a Q Exactive Plus mass spectrometer coupled to Easy nLC (Thermo Fisher, Waltham, MA, the USA). The MS data were analyzed using MaxQuant software v1.6.0.16. The results of database search were filtered and exported with <1% false discovery rate at peptide-spectrum-matched level, and protein level, respectively.

### Growth Kinetics

The rCDV-Gn and -Gc were compared with each other on their growth kinetics in VDS cells, as described previously (25). In brief, VDS cells were seeded into five 12-well plates (10^6^ cells/well, and 6 wells/plate) for incubation at 37°C for 2 h. The P15 rCDV-Gn and -Gc were separately inoculated (MOI = 0.0002) into all five plates (3 wells/progeny in each plate) for incubation at 37°C for 3 h. Supernatants were replaced with DMEM for further incubation at 37°C. A plate was randomly removed from the incubator at 0, 24, 48, 72 and 96 hpi, and then subjected to two freeze-and-thaw cycles for harvesting supernatant, followed by viral titration using the Spearman–Kärber equation (28). Growth curves of viruses were drawn using the GraphPad Prism software (Version 8.0). Data at each time point were representative of three independent experiments. As a control, the growth curve of wt-CDV referred to that in the previous publication (25).

### Genetic Stabilities of Two Foreign Sequences

Two recombinant viruses underwent twenty serial passages (72 h/passage) in VDS cells. Their culture supernatants were collected at P15 and P20 for RT-PCR detection, as described in Subheading “RT-PCR Detection”. Four RT-PCR products were subjected to agarose gel electrophoresis. Two P20 products were extracted from gels for Sanger sequencing to uncover genetic stabilities of two foreign sequences.

## Results

### Rescue of rCDV-Gn and -Gc

**Figure 2:**
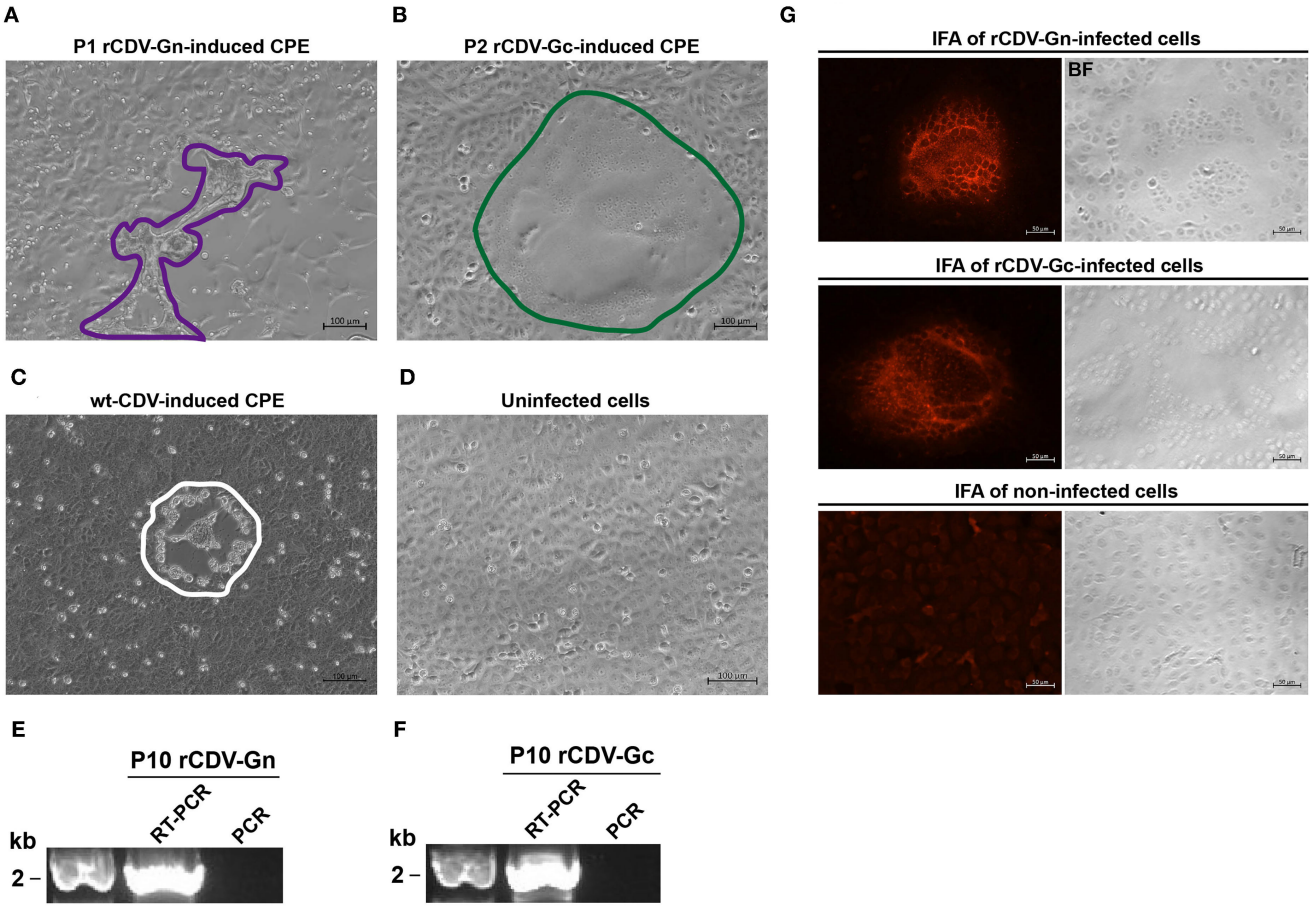
Rescue and identification of rCDV-Gn and -Gc. rCDV-Gn-induced cell-to-cell fusion [**(A)** enclosed by purple line] and rCDV-Gc-induced syncytium formation [**(B)** enclosed by green line] on VDS cell monolayers during viral passaging. As controls, wt-CDV-inoculated and uninfected cell monolayers are shown in **(C,D)**, respectively. A wt-CDV-induced CPE is enclosed by a white line. RT-PCR detection of the P10 rCDV-Gn **(E)** and -Gc **(F)** using the forward (5′-TCAAGAGTATTACTCATGCTTAA-3′) and reverse (5′-TCGAAGTCGTACACCTCAGTCAT-3′) primers. The Gn- and Gc-specific bands are 1,887 and 1,899 bps, respectively. Indirect immunofluorescence assay of VDS cell monolayers separately inoculated with rCDV-Gn and -Gc at 24 hpi **(G)**. The primary and secondary antibodies are CDV MAb and Alexa Fluor® 555 antibody, respectively.

Two full-length cDNA clones were constructed for independent co-transfection with three helpers into BSR-T7/5 cells that could constitutively express T7 RNA polymerase. Owing to the absence of CDV receptors on it, the BSR-T7/5 cell line was used only for virus recovery, rather than for blind passaging. Alternatively, the SLAM receptor-expressing VDS cells, because permissive to CDV infection, were used for serial blind passages of rescued viruses in this study. Typical cytopathic effects (CPEs), such as exacerbated cell-to-cell fusion (Figure 2A) and syncytium formation (Figure 2B), appeared on VDS cell monolayers with viral passaging. The CPEs were also visible during serial blind passages. As controls, wt-CDV-inoculated and uninfected cell monolayers were shown in Figures 2C,D, respectively.

### RT-PCR Detection of rCDV-Gn and -Gc

The rCDV-Gn and -Gc were simultaneously analyzed at P10 by one-step RT-PCR for detecting their identities. Two expected bands, 1887 (Figure 2E) and 1899 (Figure 2F) bps, were observable only on the RT-PCR lanes by agarose gel electrophoresis. As a control, PCR analysis (Figures 2E,F, Lane PCR) indicated no plasmid residue of cDNA clones affecting the RT-PCR analysis. The identities of rCDV-Gn and -Gc were confirmed by Sanger sequencing of RT-PCR products.

### IFA and Mass Spectrometry

**Figure 3:**
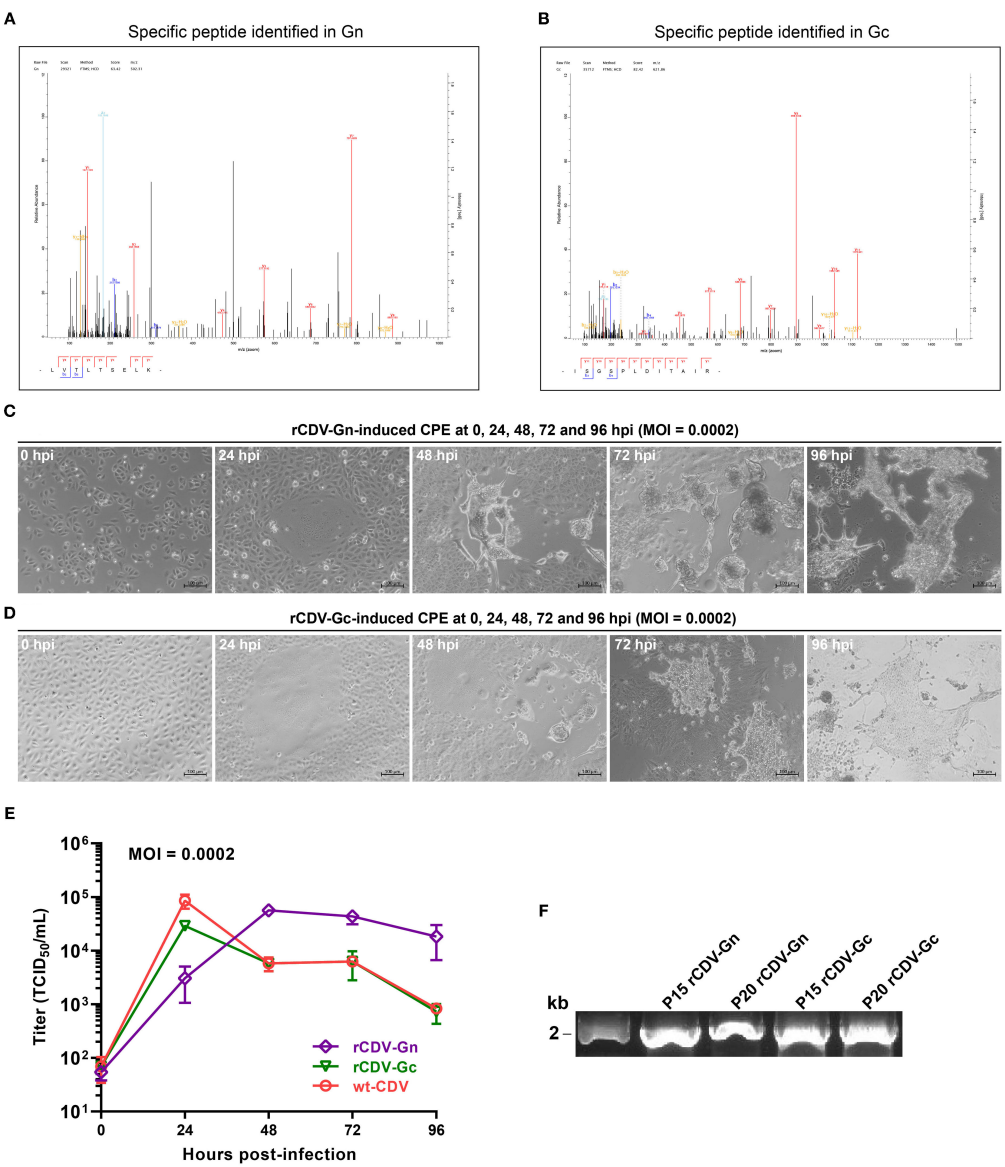
Characterization of rCDV-Gn and -Gc. Representative Gn **(A)** and Gc **(B)** specific MS/MS spectra on peptide identifications. Cytopathic effects on VDS cell monolayers separately inoculated (MOI = 0.0002) with the P15 rCDV-Gn **(C)** and -Gc **(D)** at 0, 24, 48, 72, and 96 hpi. Multi-step growth curves of rCDV-Gn and -Gc at P15 **(E)**. Three curves are drawn using the GraphPad Prism software. Data at each time point are representative of three independent experiments. RT-PCR analysis on P15 and P20 progenies of rCDV-Gn and -Gc using the forward (5′-TCAAGAGTATTACTCATGCTTAA-3′) and reverse (5′-TCGAAGTCGTACACCTCAGTCAT-3′) primers **(F)**.

In order to confirm recovery of rCDV-Gn and -Gc, the IFA was performed using CDV MAb as the primary antibody and Alexa Fluor® 555 conjugate as the secondary antibody. The result showed that bright red syncytia were visible on the rCDV-Gn- and rCDV-Gc-infected cell monolayers. As a control, non-inoculated VDS cells exhibited no similar phenotype (Figure 2G). The IFA result confirmed two recombinant CDVs had been recovered from their individual cDNA clones. Expressions of Gn and Gc were demonstrated by mass spectrometry, which exhibited Gn- and Gc-specific peptide sequences matched to the MS/MS spectra. Two representative MS/MS spectra were shown in Figures 3A,B.

### Growth Kinetics of rCDV-Gn and -Gc

To determine growth curves of two recombinants *in vitro*, VDS cell monolayers were independently inoculated with rCDV-Gn and -Gc at P15. Typical syncytium formation was visible at 24 hpi, and exacerbated over time to cause intercellular hyperfusogenicity at 48 hpi (Figures 3C,D). The growth curves of both recombinants were compared with each other and with that of the wt-CDV (Figure 3E). Two recombinants displayed distinct growth kinetics *in vitro*: the rCDV-Gn replicated more slowly from 0 to 24 hpi but maintained a higher level of titer than the rCDV-Gc did during 48–96 hpi, and approximately at 36 hpi, they showed the same titer value. The rCDV-Gc showed the similar growth kinetics to that of the wt-CDV, suggesting the Gc had a less impact than the Gn did on viral replication.

### Genetic Stability of Foreign Sequences

In order to test genetic stability of two foreign sequences, rCDV-Gn and -Gc were serially passaged in VDS cells for a total of twenty passages. The agarose gel electrophoresis showed specific RT-PCR products, separately amplified from RNA samples of P15 and P20 progenies (Figure 3F). The P20 RT-PCR products were subjected to Sanger sequencing, suggesting neither point mutation nor fragment deletion occurring in the foreign sequence of rCDV-Gn. The rCDV-Gc showed a nonsynonymous mutation (A157C) in the Gc ORF, correspondingly resulting in a mutation of amino acid (T53P) in Gc.

## Discussion

In recent years, the SFTS was frequently reported in China, Japan, and the Republic of Korea. This disease, characterized by a high case-fatality rate in humans, is primarily transmitted *via* tick bite, and can also be transmitted from person to person through contacting patient's blood (29). Domesticated animals, like companion dogs, should be considered as a source of animal-to-human transmission, as evidenced by recent case reports (7, 8, 10, 30). Unfortunately, there has been no commercially available vaccine against SFTS for dogs as yet. CDVs are efficient vectors for expressing heterologous proteins (31–34), or antigens that can confer specific immune responses in animals (22, 23, 35). This prompted us to develop a novel candidate of CDV vaccine using reverse genetics for delivering DBV antigens to induce protective immunity in dogs.

We have separately constructed two CDV reverse genetics systems for the 5804P strain (34, 36) and for the QN strain (24, 25). In the present study, we rescued two recombinant virulence-attenuating CDVs (QN strain), independently coding for DBV Gn and Gc in cells. The reason why the DBV glycoprotein precursor was not used for construction of recombinant CDV was that the full-length sequence of precursor was theoretically too long (3222 nt) to be accommodated in a single CDV genome. Even if a precursor-inserting CDV can be rescued from its recombinant cDNA clone, both viral replication and protein expression would be affected by the excessive load of heterologous sequence in a single CDV genome to some extent. Therefore, we independently rescued Gn- and Gc-expressing CDVs, in order to maintain the viral propagation that was not significantly affected by foreign sequences.

During the initial blind passages after co-transfection, both recombinants revealed a weak adaptability in VDS cells, as evidenced by slow appearance of virus-induced CPE foci (data not shown). Such a weak adaptability was gradually improved with serial passaging in VDS cells. Each viral progeny is theoretically better than its previous one in growth kinetics during the initial blind passages (37). We speculated that both recombinants had been almost adapted to the VDS cell line at P15. Thus, the P15 progenies were used for determining the growth curves of two recombinants. The rCDV-Gc had a similar growth curve to that of the wt-CDV. The rCDV-Gn showed totally different growth kinetics from those of the rCDV-Gc and wt-CDV, implying that the Gn sequence had an uncertain impact on viral replication *in vitro*. Nevertheless, Tian et al. (15) recently revealed that the insertion of DBV Gn did not affect replication of a recombinant rabies virus *in vitro*, compared with that of its parental strain. We reported a recombinant CDV (QN strain) that expressed a SARS-CoV-2 S1 subunit (686 aa) in VDS cells. The rCDV-Gn was measured to have a similar growth curve to that of the S1 subunit-expressing CDV (25). More recently, we rescued another recombinant CDV (QN strain) that could efficiently express the VP2 of canine parvovirus type 2. Interestingly, we found the VP2-expressing CDV showed also a similar growth curve to that of the rCDV-Gn (24).

To enhance expression levels of Gn and Gc, their full-length ORFs were optimized for codon usage bias in dogs. Their expressions were qualitatively analyzed by mass spectrometry, demonstrating that the rCDV-Gn and -Gc were able of encoding the Gn and Gc in VDS cells, respectively. It is generally assumed that bunyaviral Gn and Gc induce specific immune responses *in vivo* (11–14). Much research remains to be performed to reveal whether both the rCDV-Gn and -Gc can elicit specific immunity in animals.

The CDV Rockborn strain, albeit historically regarded as a virulence-attenuating one, reverted back to a highly virulent status after serial passaging in dogs (38). Therefore, the viral feature of high-fidelity replication plays a crucial role in development of live-attenuated CDV vaccines, and ensures a foreign antigen stably expressed for inducing repeatedly immune responses *in vivo*. In the present study, we hoped to rescue two recombinant CDVs, characterized by high-fidelity replication during serial passages. The Gn ORF was demonstrated to be genetically stable at P20, whereas unfortunately the Gc ORF showed one missense mutation (A157C). We recently reported a recombinant CDV (5804P strain) that could express enhanced green fluorescence protein (eGFP) in cells. Under non-selective conditions, this eGFP-tagged recombinant exhibited only one single-nucleotide mutation in the eGFP ORF at P47 (36). We have successfully established the reverse genetics systems of two CDV strains. Although it remains to be clarified which strain has a higher fidelity in viral replication, the QN is more suitable than the 5804P for use as a vector candidate, because the former has been proven to be a virulence-attenuating strain (unpublished data), whereas the latter is a highly virulent one (39).

## Data Availability Statement

The original contributions presented in the study are included in the article/supplementary material, further inquiries can be directed to the corresponding author/s.

## Author Contributions

JL, YL, LL, QW, and HZ performed the experimental works. FL and BN conducted experiments, wrote the manuscript and provided fundings. All authors read and approved the final manuscript.

## Funding

This work was supported by the Research Foundation for Distinguished Scholars of Qingdao Agricultural University (1120045), and by the Innovation Fund of China Animal Health and Epidemiology Center.

## Conflict of Interest

The authors declare that the research was conducted in the absence of any commercial or financial relationships that could be construed as a potential conflict of interest.

## Publisher's Note

All claims expressed in this article are solely those of the authors and do not necessarily represent those of their affiliated organizations, or those of the publisher, the editors and the reviewers. Any product that may be evaluated in this article, or claim that may be made by its manufacturer, is not guaranteed or endorsed by the publisher.
